# Integration of HIV and cervical cancer screening perceptions of healthcare providers and policy makers in Uganda

**DOI:** 10.1186/1471-2458-14-810

**Published:** 2014-08-07

**Authors:** Edward Kumakech, Sören Andersson, Henry Wabinga, Vanja Berggren

**Affiliations:** School of Health and Medical Sciences, Örebro University, Örebro, Sweden; Department of Pathology, Makerere University, College of Health Sciences, Kampala, Uganda; Department of Laboratory Medicine, Örebro University Hospital, Örebro, Sweden; Division of Health Sciences, Lund University, Lund, Sweden

**Keywords:** HIV, Cervical cancer, Screening, Integration, Uganda

## Abstract

**Background:**

HIV-positive women have an increased risk of developing cervical cancer (CC) compared to the HIV-negative women. Despite this, HIV and CC screening programs in many developing countries have remained disintegrated. Therefore, the objective of the study was to explore perceptions of healthcare providers (HCP) and policy makers (PM) about integration of HIV and CC screening services in Uganda.

**Methods:**

This was a qualitative study conducted among 16 participants comprising of 12 healthcare providers and 4 policy makers in Uganda. Data were collected through individual interviews. Participants were purposively selected from different level of health facilities with clinics for HIV and CC screening services. Content analysis method was used to analyze the data.

**Results:**

Three themes emerged from the data, namely appreciating benefits of integration, worrying about the limited health system capacity and potential consequences of integration and feeling optimistic about integration under improved health system conditions. The benefits embraced the women – particularly the HIV-positive women- but also men, healthcare providers and the health system or the government. There were worries that HIV stigma and shortage of healthcare workers would affect the effective delivery of the integrated program.

**Conclusion:**

Integration of HIV and CC screening can offer manifold benefits to all stakeholders in the health system, more so to the women. However, its feasibility in developing countries such as Uganda will most likely be hampered by weak and inefficient health systems. Therefore, when considering HIV and CC screening integration, it is important not to only recognize the benefits but also take into account resources requirements for addressing the existing weaknesses and inefficiencies in the health systems such as limited infrastructure, insufficient drugs and supplies, inadequate and poorly motivated healthcare workers.

## Background

Cervical cancer (CC) is the second most common cancer among women globally, with an estimated 529,409 new cases and 274,883 deaths in 2008 [1]. About 86% of the deaths occur in developing countries, making it the leading cause of cancer death among women [2]. In Uganda, CC is the commonest cancer among women. The age-standardized incidence rate for CC is on the increase from 17.7/100,000 women person years in early 1960s to 47.5/100,000 women person years in 2008 which is one of the highest in the world [3, 1].

HIV-positive women have an increased risk of developing CC compared to the HIV-negative women [4–8]. In view of the above, the World Health Organization (WHO) recommends more aggressive re-screening schedule for HIV-positive women compared to HIV-negative women [9]. More so, HIV and human papillomavirus (HPV), the cause of CC [10, 11] are both sexually transmitted infections (STIs) and so share the same risk factors such as multiple sexual partners [12–14].

In view of this, interventions for HIV and CC prevention such as screening should ideally be delivered in an integrated approach. And recent studies concluded that there is a large potential health gain if HIV and CC prevention services are integrated [15, 16]. Policy makers (PM)s from disciplines outside the cancer domain—for example HIV care — are warranted to create CC prevention services for women attending that programs/facilities [16].

Recent studies have also demonstrated various models of integrating HIV and CC screening services including information on their effectiveness and acceptability among the HIV-positive women. For example, in Zambia where CC prevention clinics were co-located within the public health clinics offering HIV/AIDS care and treatment, manifold advantages were observed including resource and infrastructure sharing, availability of a wider range of women’s health services for HIV-infected/at-risk women, and opportunities for referral between the clinic systems and maximization of participation in both programs [17]. The above study also pointed out that stigma associated with HIV/AIDS somehow affected the uptake of the integrated services. Similarly, in Kenya were CC screening services were integrated into mother, child health (MCH) and family planning (FP) clinics, it was reported that a high proportion of women who visited the MCH-FP clinics for well-baby or family planning services also benefited from CC and sexually transmitted infections (STIs) screening and treatment services in a single visit approach [18]. In Nigeria, where women seeking reproductive health (RH) or HIV care services were bi-directionally referred to either HIV care or RH clinics co-situated within the same health facility, it was demonstrated that CC screening by visual inspection with 5% acetic acid (VIA) was highly acceptable and facilitated early detection and treatment of very many cases of cervical pre-cancerous lesions among women [19, 20].

Despite the above evidence and recommendations, in Uganda and many other developing countries, HIV and CC screening services continue to be implemented as standalone programs. As disintegrated clinics, almost all the HIV care programs in Uganda do not offer CC screening services and hence the female HIV patients miss CC screening opportunities despite the frequent visits they make to the HIV facilities for reviews and drug refills. This increases their risk of presenting late with advanced CC disease with poor prognosis. Similarly, almost all CC screening programs in Uganda do not offer HIV screening services to women and hence the women risk receiving inappropriate schedules for CC re-screening. Less aggressive CC re-screening interval for HIV positive women similarly increases the risk of them presenting late with advanced CC disease with poor prognosis.

Therefore, the objective of this study was to explore the perceptions of healthcare providers (HCP) and PM perceptions regarding integration of HIV and CC screening services in Uganda.

## Methods

### Study design

This was a qualitative study. It was chosen to allow for in-depth exploration of perceptions of the integrated approach to delivery of HIV and CC screening services, the phenomenon under investigation [21, 22].

### Study sites

The study was conducted at CC clinics in 4 districts in Uganda namely Kampala, Mbarara, Ibanda and Nakasongola but the participants were drawn from 9 clinics of 7 health facilities and 4 district health offices within the 4 districts. Table 1 shows the clinic characteristics. These districts and clinics provided perspectives of urban, semi-urban, rural and remote settings of Uganda. More so, these study sites provided the perspectives of the different levels of healthcare in Uganda in addition to public and private health sectors perspectives.

**Table 1:** **Characteristics of participating clinics and district health offices**

Sn	District	Region	Ownership	Location	Services
1	Kampala	Capital city	Public	Urban	VIA, PNC and FP
2	Kampala	Capital city	Public	Urban	VIA, Cryo, Colpo and LEEP
3	Kampala	Capital city	Private	Urban	VIA, HIV testing, ART
4	Kampala	Capital city	Public	Urban	VIA and cryotherapy
5	Kampala	Capital city	Private	Urban	HIV testing and treatment
6	Kampala	Capital city	Public	Urban	Health policies
7	Mbarara	Western	Public	Semi-urban	VIA, Cryo, Colpo and LEEP
8	Mbarara	Western	Public	Semi-urban	HIV testing and treatment
9	Mbarara	Western	Public	Semi-urban	Health policies
10	Ibanda	Western	Private	Rural	VIA and Cryotherapy
11	Ibanda	Western	Public	Rural	Health policies
12	Nakasongola	Central	Public	Rural	VIA and cryotherapy
13	Nakasongola	Central	Public	Rural	Health policies

VIA is visual inspection with 5% acetic acid solution; PNC is postnatal care; FP is family planning; LEEP is Loop Electrosurgical excision procedure; Cryo is Cryotherapy; Colpo is Colposcopy.

At the time of the study, all the study clinics have implemented CC screening services for at least 3 years. And the CC screening services they provided included health education talks, counseling, CC screening by VIA, treatment of cervical precancerous lesions using cryotherapy, referral for CC diagnosis, staging and treatment and reviews of women after cryotherapy. More so, all the study clinics were not implementing an integrated delivery of HIV and CC screening services with an exception of only one private facility situated in an urban setting. And therefore, the HIV status of the women were established through history and those who have never tested for HIV were presumed and in most of the time treated as HIV negative women.

### Selection criteria for study sites

Seven health facilities were purposively selected to participate in the study. A health facility is eligible for selection to participate in the study if it is situated in any of the four study sites namely Kampala, Mbarara, Ibanda and Nakasongola districts and has clinics that provide HIV and CC screening services.

### Study participants and sample size

Sixteen participants were selected for the study. They were 12 healthcare providers and 4 district level policy makers. The healthcare providers included medical doctors, nurses, midwives and clinical officers with experience in delivery of both HIV and cervical cancer screening services. The demographic characteristics of the participants are shown in Table 2. Being a qualitative study, the sample size of 16 participants was determined by data saturation point, a point at which further sampling doesn’t generate any new concepts about the phenomenon under investigation.

**Table 2:** **Characteristics of study participants**

PID	Age	Sex	Occupation	Health facility address	HIV work	CC work
1	53 yrs	F	Nurse	Public HC in urban area	05 yrs	12 yrs
2	47 yrs	F	Nurse	Public HC in urban area	03 yrs	12 yrs
3	38 yrs	F	Nurse	Private MC in urban area	08 yrs	04 yrs
4	25 yrs	F	Nurse-midwife	Public RRH in semi-urban	03 yrs	04 yrs
5	54 yrs	F	Midwife	Public NRH in urban area	05 yrs	05 yrs
6	35 yrs	M	Medical doctor	Public HC in rural area	06 yrs	03 yrs
7	39 yrs	F	Gynecologist	Public NRH in urban area	03 yrs	12 yrs
8	41 yrs	M	Clinical officer	Public HC in rural area	15 yrs	03 yrs
9	40 yrs	F	Midwife	Public HC in rural area	08 yrs	02 yrs
10	38 yrs	M	Nurse-midwife	Private CRC in urban area	09 yrs	01 yr
11	30 yrs	M	Nurse	Public RRH in semi-urban	05 yrs	01 yr
12	30 yrs	F	Nurse	Public RRH in semi-urban	01 yr	01 yr
13	32 yrs	F	Nurse-midwife	Private ASO in rural area	05 yrs	01 yr
14	46 yrs	F	Midwife	Public NRH in urban area	03 yrs	10 yrs
15	29 yrs	F	Nurse-midwife	Public HC in rural area	05 yrs	03 yrs
16	43 yrs	F	Midwife	Private GH in rural area	12 yrs	04 yrs

Key: PID is participant identity; F isfemale; M is male; CC is cervical cancer; yr(s) is year(s); NRH is National Referral Hospital, HC is Health centre; MC is Medical centre; RRH is Regional Referral Hospital; GH is General Hospital; ASO is AIDS Support Organization; CRC is Clinical Research Centre; participants 6–8 and 14 were holding job positions of policy makers.

### Study field team and procedures

The study field team comprised of the first author (EK), the last author (VB), four healthcare providers and four district health officers (one from each study site). The healthcare provider and district health officer at each study site provided the list of names and contact addresses of all the healthcare providers and policy makers working in HIV and CC screening clinics respectively. The lists were then used as sampling frames to select participants who fulfilled the eligibility criteria. All the interviews were conducted by the first (EK) and last authors (VB).

### Sampling method for participants

The participants were selected using purposive sampling method [23]. This sampling method allowed for selection of participants with experience in delivery of both HIV and CC screening services.

### Participants’ recruitment procedure

The researcher and the team visited each selected study site to develop a list of eligible participants for each study site. The researcher informally asked the head of the cervical cancer clinic in each study site regarding the HIV and CC screening work experiences of each of the potential participant in their department. Policy makers and healthcare providers with at least 6 months of work experience in both HIV and CC screening clinics were eligible to participate in the study. Based on this criterion, a range of 1–2 policy makers and 2–4 healthcare providers per study site were eligible to participate in the study. And their work experience in HIV and CC screening clinics ranged from 6 months to 12 years. Eligible participants with at least 1 year of work experience in both HIV and CC screening clinics were first selected and invited to participate in the study. The selected participants were invited to participate in the study through telephone call. Meeting appointments were made with participants who accepted to participate in the study. All the 4 policy makers selected and called by telephone accepted and gave appointments to participate in the study. As for healthcare providers, 13 were selected and called by telephone, 12 accepted, 1 refused to participate in the study. The one who refused was from a private medical centre in Kampala capital city and her reason for refusal was lack of time to take the interviews within the data collection period.

### Data collection method

Data were collected between February 2012 and February 2013. Individual interviews (IDIs) were used to collect the data. Individual interviews was a data collection method of choice for addressing the research question of this study (i.e. perceptions of HCPs and PMs regarding HIV and CC screening integration) because it has greater potential of allowing participants to freely share sensitive personal opinions such as beliefs, attitudes and prejudices which are more likely to be withheld in focus groups. The interviews were conducted by the first and last authors in English language because being healthcare providers and policy makers, all the participants were fluent in English language. All the IDIs were conducted in a quiet environment outside the participant’s workplace to avoid disruption from patients and other visitors to their workplaces. All the interviews were recorded using an audio-recording device. A separate sheet of paper (one for each interview) was used to record the socio-demographic data of each participant. Participants’ names were not recorded or written anywhere to ensure confidentiality. All the interviews lasted for utmost 90 minutes.

### Interview guide

The instrument used for data collection was a semi-structured interview guide. The interview guide was specifically developed for the study and had items aimed at eliciting participants’ feelings, opinions or views about integrated delivery of HIV and CC screening services to women in a single visit approach. The items in the interview guide were constructed based on theory of change and phrased in such a way to allow participants to examine and share their perceptions of the four quality control criteria of theory of change namely plausibility, feasibility, testability and appropriate scope [24]. And some of the key questions in the interview guide include what were the participants impressions of HIV and CC screening services integration in single visit approach, what could be the advantages and disadvantages of integration to the women, men, healthcare providers, health system and funders of healthcare programs such as government, would integration be feasible, acceptable and appropriate in public and private health systems in low income country such as Uganda.

### Data analysis

**Figure 1:**
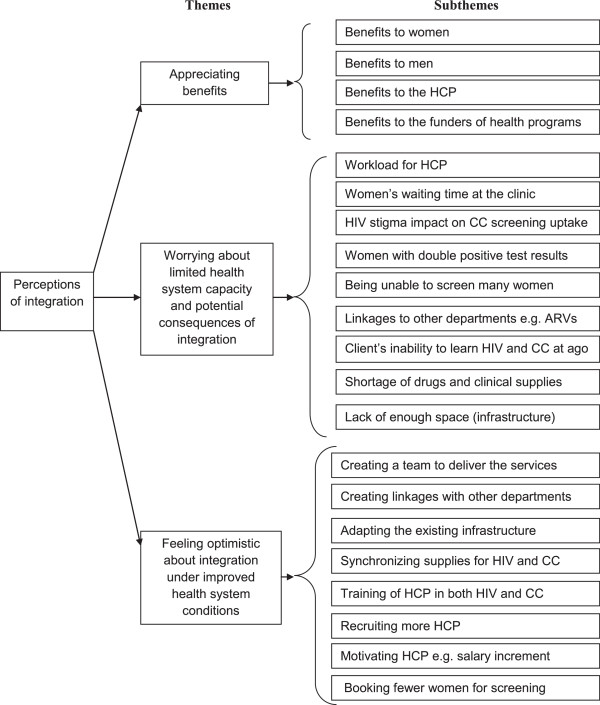
**Showing themes and subthemes.**

Data (in audio-recording device) were first transcribed verbatim and then analyzed using content analysis method [25]. The content analysis method allowed for exploration and synthesis of codes, themes and categories. Figure 1 shows the code list together with the corresponding themes and categories that were developed for the entire data.

### Rigors of the research

Rigors of this qualitative study was ensured by paying attention to the credibility, trustworthiness and transferability of the research [25]. The researcher’s prolonged engagement with the data both during the collection and analysis phases ensured credibility of the data. To ensure trustworthiness of the study, several categories of participants also known as triangulation of data sources and also two researchers participated in data collection and analysis also known as triangulation of researchers [23]. Furthermore, to ensure transferability of the study, the setting and context of the study were explicitly described in the study sites section [25].

### Ethical considerations

Ethical approval for the study was obtained from Institutional Review Board (IRB) of School of Biomedical Sciences Makerere University College of Health Sciences in Uganda and also from the Uganda National Council for Science and Technology. Informed consents were obtained from each participant before data collection.

### Reporting guidelines

This manuscript is adherent to the RATS guidelines for reporting qualitative studies which stands for relevance of the study question (R), appropriateness of the qualitative method (A), transparency of procedures (T), soundness of the interpretive approach (S) [26]. This is to ensure compliance with internationally acceptable standards for reporting qualitative studies.

## Results

From the data analysis, three themes emerged about integration of HIV and CC screening services. These were appreciating benefits, worrying about the limited health system capacity and potential consequences of integration and feeling optimistic about integration under improved health system conditions.

### Appreciating benefits of integration

All the participants viewed the integrated approach to delivery of HIV and CC screening services will provide benefits. The benefits mentioned were categorized as multiple benefits to the women, economic benefits to men, a few benefits to HCP and economic benefits to the health system as a whole.

### Multiple benefits to the women

All the participants felt that an integrated approach to delivery of HIV and CC screening services would provide the women with the opportunity to receive more health services including information in a single visit to the health facility. The participants explained that through integration, HCP will inform and offer women the chance to receive all the available health services whenever they come to the integrated clinic. One participant said,

*“With integration you can hit two birds with one stone. CC screening especially in rural areas, people don’t know anything about it but they know things about HIV and they come for HIV testing. So if they come for HIV testing, it’s an opportunity to tell them that you also need to screen for CC and vice versa” – (HCP 6).*

Almost all the participants thought that integration will increase access to CC screening services among the HIV positive women, who are the rightful target for the service because of their increased risk of developing CC compared to the HIV negative women. The participants thought this is possible because the HIV positive women are frequent visitors to the health facility for HIV services particularly antiretroviral (ARV) drugs refills and so chances are high that they would use one of those visits to seek CC screening services, if integrated within the HIV care program. One participant said,

*“You are going to capture a very good number of women especially the HIV positive women who are usually told to return regularly to the health facility for reviews and ARV drugs refills. Clients are going to be very many than when you specialize” – (HCP 1).*

Almost all the participants thought that integration will prevent HIV positive women on ARV drugs dying from CC instead. One participant explained this by saying that if the CC screening aspect is ignored for the HIV positive women, it can instead turn out to be the cause of death of HIV/AIDS patients even though they were put on ARV drugs, because the CC risk, disease progression and severity is higher in the HIV-positive population compared to the HIV-negative population. Another participant said,

*“Actually, every HIV care facility should screen women for CC because actually women may die from CC instead of the HIV they are treating” – (HCP 6).*

Almost all the participants thought that integration will reduce on the frequency of visits women make to health facilities for health services. The participants thought this to be an economic benefit to the women because it will enable them to save on travel time and cost to health facilities for health services. One participant said,

*“…. It will be money saving and time saving, so the people who live far away from the health facility will be the one who will benefit most” – (HCP 12).*

One participant thought that integration will help to reduce on the worry women often get when they are referred from CC clinic to go elsewhere for HIV testing services.

Almost all the participants thought that integration will facilitate early detection and treatment of other diseases especially gynecological diseases that the women may not even be aware of. The participants viewed this as a benefit to the women because through integration they will receive a holistic treatment of their disease conditions. One participant said,

*“From experience we have treated so many sexually transmitted infections that we used not to identify before integrating CC and HIV screening” – (HCP 3).*

Some participants thought that integration would enable HCP to detect and treat CC lesions at an early stage and save many women especially the HIV positive women from getting to the late stages of the disease. One participant said,

*“I do like the idea of integration so much because the period the HPV infection takes to progress to invasive cervical cancer is shorter in HIV clients so if it’s integrated it would save many people from getting to the last stages of the CC disease” – (HCP 4).*

Many participants thought that integration will help to minimize loss to follow up of women from CC screening program. One participant from a private health facility with experience of integration explained that this can happen if HCP working in the integrated clinic try as much as possible to match the follow up dates for CC with that of ARV drugs refill so that when the woman returns to the health facility for CC review she can also get ARV drugs refill. Another participant said,

*“I think integration will be a good idea. The client will not miss either of their appointments” – (HCP 4).*

All the participants from CC clinics thought that integration would enable HCP to give proper or correct follow up schedule for the HIV positive women who turn out to be positive for cervical pre-cancerous lesions e.g. setting up an aggressive follow up schedule usually after every 1 year instead of after every 3 years for the HIV negative women. One participant said,

*“I think integration will be a good opportunity because …, reason being the way we manage those who are HIV positive with cervical lesions, their follow up has to be aggressive. While for her (the HIV positive woman) we tell her you need to come back after 1 year and yet we would have told her to come back after 3 years if she were HIV negative” – HCP 7.*

Almost all the participants thought that HIV and CC screening integration will increase the availability of CC screening sites for women. The participants explained that HIV testing sites are very many, almost in all health facilities countrywide and if each of them integrates CC screening services then the number of CC screening sites will also increase countrywide, making the CC screening services widely available to women. One participant explained,

“*Integrating HIV and CC screening will be a very good check, it will at least increase community awareness about the CC screening and its availability definitely will also increase” – (HCP 16)*

### Knowledge and skills benefits to the HCP

A few participants thought that integration will enable HCP to acquire more knowledge and skills in delivery of both HIV and CC screening services. One participant said,

*“…. maybe acquiring the skills of how to screen for HIV which most of them have been trained in but currently in our CC clinic we are not doing it” – HCP 7.*

Similarly, a few participants thought that integration will ease work for HCP as it will enable them to make correct diagnosis and proper treatment for the women, which will in the long run save the HCP time for seeing the same woman again for the same complains. One participant said,

*“I think even us health workers, it ease our work because you will get rid of many of the diseases you have to treat. You don’t only provide ARV drugs for HIV treatment but you also provide these other drugs anti-fungal, antibiotics. Many times they (women) come to the health facility thinking that it’s maybe STIs, urinary tract infections (UTIs) or pelvic inflammatory diseases (PID). You think you are treating a PID when it’s actually CC. So I think it helps us to diagnose a client well before giving the treatment” – (HCP 4).*

### More than economic benefits to men

It further emerged that participants of this study viewed integration will also provide some benefits to men.

Almost all the participants thought that integration will also enable men save both on transportation time and cost of their female partners to the health facility for health services. A few of the participants shared their observation that integration has in fact encouraged male involvement in reproductive health of women especially their female partners. One participant said,

*“The husbands will accept it because of the transportation issue. They will actually like the idea that they only have to pay one transport cost for both screening so it will be both time and cost saving to them, instead of paying this week for one test and then next week go for the other tests ” – (HCP 12).*

Another benefit of integration to men expressed by a few of the participants of this study was the possibility that men who accompany the women to the health facility would themselves utilize some of the services such as listening to health education talks, HIV counseling and testing from the integrated clinic.

### Economic benefit to the health system

Most of the participants thought that integration will provide economic benefits to the health system as a whole. Almost all of them mentioned that integration will reduce the health expenditure in that it will be less expensive for the health system to deliver services in an integrated approach. One participant explained,

*“It also reduces on the expenditure of the health system because instead of health workers going and making an outreach for HIV and making another outreach for CC screening, you have an opportunity to do both of them on the same client on the same day which is less expensive for the health system” – (HCP 6).*

### Worrying about limited health system capacity and potential consequences of integration

Almost all the participants felt worried that HIV testing may instead become a barrier to uptake of CC screening services especially among women who do not want to take HIV test because of fears of HIV stigma. One participant said,

*“The disadvantage of integration is that people associating CC screening with HIV testing. People may fear to come for the CC screening knowing that if you go to the screening, they will also test for HIV. There are some people who may fear” – (HCP 6).*

Similarly, all the participants felt worried that integration will increase the workload for HCP which will subsequently affect their efficiency and productivity. One participant said,

*“With integration, the HCP will have a lot to do. They will have the double workload of dealing with both HIV testing and CC at the same time” – (HCP 12).*

Furthermore, a few of the participants felt worried that integration would make HCP to screen very few women per day. One participant said,

*“But I think with integration, we can only serve 5 clients in a day, but the others who came but weren’t served will be offended” – (HCP 12).*

### Feeling compassion for the women

A few of the participants felt compassion for the women will turn out to be double positive i.e. positive for both HIV and CC lesions because they thought it would make them more miserable. One participant said,

*“Sometimes they (women) don’t want to also know their HIV status because they say why should I know now, it will make me more miserable if also found positive” – (HCP 7).*

Similarly, almost all the participants felt compassion for the women whose waiting time at the health facility would be prolonged due to the integrated approach because they thought it would offend them. One participant said,

*“The only disadvantage to the woman maybe is the longer waiting time because before we screen her we are going to have a counseling session, then she will be screened for HIV, then she will wait for the results of the HIV test and then she will again be screened for CC, maybe more time at the Clinic that’s the only disadvantage to the women” – (HCP 7).*

More so, a few of the participants also felt compassion for the women who will be unable to comprehend both concepts of HIV and CC screening services that will be presented to them in an integrated format.

### Feeling optimistic about integration under improved health system conditions

There were positive feelings among almost all the participants that integration would be feasible under improved conditions. And conditions were categorized as improving resources and minimized waiting time for women at the health facility.

### Improved resources

Almost all the participants emphasized the importance of recruiting more HCP to do the extra work that will be created by the integration. One participant said,

*“Integration is possible only if extra healthcare staff is provided” – (HCP 12).*

Also many participants expressed their view that for integration to be effectively implemented every supplies required including drugs have to be made available. One participant said,

*“HIV clients have many gynecological diseases such as cervicitis, bleeding all the time, cervical warts, you have to be equipped with drugs” – (HCP 2).*

A few participants thought that integration can work if the existing facilities can be adapted to create space for counseling, CC screening, treatment and then the HIV testing. One participant said,

*“Where they are having more place everything should run in the same place, you can put there cancer screening, HIV screening, the treatment, everything, you proceed…” – (HCP 5).*

More so, all the participants expressed their view that HCP should be trained in both HIV and CC screening to acquire the necessary skills to provide the services in an integrated manner. One participant shared how being trained in both HIV and CC screening helps in integration,

*“If they come to the CC clinic, us who do the cancer screening are also trained in Antiretroviral therapy (ART) so you do the CC screening and also write them the ARV drugs so they go because one of the things they did was to take us for training in the ART, so that saved a lot on their waiting time at the facility” – (HCP 4).*

### Minimized waiting time for women

Almost all the participants thought that integration would work if effective strategies are put in place to minimize women’s waiting time at the health facility.

Most of the participants felt that in order to address the issue of long waiting time for women at the integrated clinic, a team of HCP should be put in place to perform the different tasks in the integrated clinic. One participant said,

*“it shouldn’t be the same HCP to provide both the HIV and CC screening reasons being, you need to put on gloves, touching genitals of the woman and for HIV testing you are touching blood, you need to be different people so that you are fast” – (HCP 2).*

A few participants expressed their view that there is need for the integrated clinic staffs to collaborate with the laboratory unit personnel who run the HIV tests so as to speed up the HIV testing procedure. One participant said,

*“We have to collaborate with the laboratory people, for you will take the blood and finish CC screening in 2–3 minutes but the HIV results will delay in the laboratory, at least the laboratory personnel should take 20–30 minutes” – (HCP 1).*

More so, a few participants expressed their view that the strategy should be to book a few women per day for the integrated screening. One participant said,

*“But we try to handle that (delay of clients in queue) by booking a few clients per day. We can have like 10 women to screen on one day to reduce on the waiting time. I personally have not found any challenge with it (integration). First of all, I book specific number of clients I will see that day. If I have 10 people, I work on that for my day” – (HCP 4).*

Some participants expressed their view that all the HCP with the required expertise should be put to work together whenever possible in the same room. One participant said,

*“We had the issue of long waiting time initially, but we solved it by integrating everything in the same room. When someone comes, there is a doctor to see them; there is another HCP to screen them for CC at the same point, not allowing them to go to stand in several queues at different service points. By the time the client leaves the clinic, they just go to the pharmacy to collect their prescribed drugs” – (HCP 3).*

Furthermore, almost all the participants thought that a non-mandatory approach to integration should be adopted in order to allay fears of long waiting time and not to chase away women who may not be ready to receive both tests in a single visit and also to dissociate CC screening from HIV testing. One participant said,

*“….. It’s very important that we don’t make it compulsory. We should openly tell them that they are free, they can decide to take both or one test……………… I think the important thing is to make it optional, give them the information but leave it optional so that people don’t connect HIV testing to CC screening”– (PM 4).*

## Discussion

One of the key findings of this study was the view by HCP as well as PM that integration of HIV and CC screening would provide benefits to all the concerned stakeholders. This included benefits to the women, to the men, to healthcare providers and to the health system. More so, HIV and CC integration was viewed to provide more benefits to the women than to any of the other stakeholders. The manifold benefits of an integrated approach to HIV and CC screening expressed by participants of this study concurs with those observed by Mwanahamuntu et al., [17] in Zambia. In both of these studies, the benefits mentioned included resource and infrastructure sharing, availability of a wider range of women’s health services for HIV infected/at-risk women, and opportunities for referral between the clinic systems and maximization of participation in both programs. These similarities of study findings could be an indication that stakeholders across Africa share a common view about the benefits of an integrated approach to HIV and CC screening.

Notably, there were many other benefits of integration that were expressed by participants of this study which were not expressed in previous studies. This includes the view that HIV and CC screening integration would reduce on the frequency of visits women would make to health facilities for healthcare services, travel time and cost saving to the women and their male partners, minimized loss to follow up of women scheduled for HIV and CC treatment, and correct or proper management of HIV positive women with cervical precancerous lesions. These additional benefits of HIV and CC screening integration if incorporated into community education programs could provide motivations for women to seek HIV and CC screening.

The view by the participants of this study that HIV and CC screening integration would provide opportunity for women to receive early detection and treatment for other gynecological diseases such as STIs concurs with Were et al., [18] findings in Kenya regarding CC screening integration into MCH and FP clinics. The benefits of detecting other gynecological diseases in the woman is irrespectively derived from the visual methods as a CC screening method because the procedure allows the HCP to visualize the genitalia of the women for signs of gynecological diseases. We think this benefit of detection of other gynecological diseases is less likely to be realized if other CC screening methods rather than the visual methods were used in the integration.

Notably, this study revealed that despite the general consensus among almost all categories of the participants that HIV screening programs in Uganda are more widespread and have better coverage than CC screening, there were conflicting views on whether integration would be of benefit to women who do not attend HIV centres because of the fear of HIV/AIDS stigma or they would similarly become reluctant to attend CC clinics where HIV testing is also offered. Future research should address this question as previous HIV/AIDS stigma studies haven’t really examined whether HIV/AIDS stigma diminishes with the type of clinic.

The participants of this study thought that integration of HIV and CC screening would also provide economic benefits to men as well as encourage reproductive health seeking behaviors among them. This finding is logical because in some Ugandan cultures, men are responsible for the medical bills of their female partners and often transport or accompany the women to health facilities and attend the health talks about HIV or CC provided by the HCP. After attending the health talks, it is possible that some men can decide to seek for health services for themselves, e.g. HIV screening and therefore attain more than economic benefits from the integration.

Another finding of this study was the perception by the participants that HIV and CC screening integration would provide HCP with the opportunity to acquire more knowledge and skills in both HIV and CC screening services. It is logical that HCP will acquire more experiential knowledge and skills as they practice from the integrated clinic. The unanswered question is whether this perceived benefit (acquiring more knowledge and skills) outweighs the perceived challenge of integration (e.g. increased workload) to act as a cue to the action of integration among HCP.

More so, the view of the participants of this study that integration would reduce on the health expenditure concurs with that of Mwanahamuntu et al., [17] in Zambia that integration allows for sharing of resources and infrastructure between HIV and CC programs, which in a way reduces the health expenditure. This particular finding about the potential of integration to leverage resource sharing between HIV and CC programs could be incorporated into advocacy materials targeting Ministry of Health officials, planners and policy makers.

Furthermore, consistent with previous studies was this study finding that there were worries among almost all the participants that HIV stigma would affect uptake of CC screening services from an integrated clinic. The same worry was expressed about HIV and CC screening integration in Zambia by Mwanahamuntu et al., [17]. This implies that across African countries, HIV stigma is still a reality and continues to deter some people from accessing HIV services despite the huge investment in HIV/AIDS programs. And therefore, strategies for dissociating HIV testing from CC screening such as opt-out options should be in-built in all informational or educational materials about integrated HIV and CC screening services. Efforts should also be made to reduce HIV/AIDS-related stigma.

Also consistent with previous studies was this study finding that almost all the participants expressed their worry that HIV and CC screening integration would increase workload for HCP. Increased workload was thought to lead to negative consequences such as screening fewer women per day, prolonged waiting time for women at the health facility and failure to screen all and hence disappointment of some women who have come to the clinic for screening. The same worry was expressed in another African country, Mozambique, where a pilot CC screening was implemented among women attending HIV care program [27]. The similarity in the findings from the two studies can be explained by the shortage of HCP which is a problem in both countries and many other African countries.

The study finding that almost all the participants felt optimistic about the feasibility of integrating HIV and CC screening under improved conditions of the health system is also consistent with the previous study in Mozambique [27]. The study in Mozambique recommended for improvements in the health infrastructure to create room for CC screening integration into HIV care facilities.

Although the use of HPV DNA testing on self-collected vaginal samples as one of the CC screening algorithm options for integration with HIV testing didn’t emerge from the study but because of its potential in addressing some of the perceived challenges of integration observed in this study such as delay of women at the integrated clinic and increased workload for HCP makes it worth discussing. With HPV DNA testing on self-collected vaginal samples as the CC screening algorithm of choice, women could do self-sampling from home or laboratory and pass via the laboratory for HPV DNA and HIV testing first. And those with positive test results could proceed to the integrated clinic for pelvic examination using visual inspection with acetic acid (VIA) and treatment. The HPV DNA testing with self-sampling for CC screening algorithm has the advantages of increasing CC screening coverage among women, minimizing the delay of women at the health facility and evenly dividing the workload created by integration among laboratory and clinic HCP. The disadvantage would be that women with HPV DNA negative test results will miss pelvic examination with visual methods plus its other benefits such as detection and treatment of unsuspected STIs by the HCP at the integrated clinic.

### Study limitations

Just like all studies, this study is not without limitations. The qualitative nature of the study couldn’t allow large sample size and consequently the study findings are not meant to be generalizable. More so, the use of interview method of data collection for this study carries the risk of response bias – that is the tendency of some respondents to distort their responses and the most pervasive being the tendency to present a favorable image of the phenomenon under investigation [28]. Response bias was minimized in this study by the interviewer being aware of the many ways the results can inadvertently be biased by the interview as a data collection method and bracketing them during the interview. Worse still, the use of qualitative content analysis for data analysis suffers from the risk of subjectivity [28]. This risk of subjectivity was minimized by two of the investigators first analyzing the data independently and then jointly to reconcile differences in the interpretation of the data, themes or categories. Nevertheless, in view of the above limitations of interviews and content analysis methods employed in this study, the results and conclusions should be interpreted and used within the context of Uganda or similar healthcare system in low resource settings unless otherwise.

## Conclusions

Integration of HIV and CC screening can offer manifold benefits to all stakeholders in the health system, more so to the women. However, its feasibility in developing countries such as Uganda is likely to be hampered by weak and inefficient health system characterized by limited infrastructure, insufficient drugs and clinical supplies, inadequate and poorly motivated HCP. HIV stigma continues to linger as a stumbling block that can potentially affect access to services from an integrated HIV and CC screening program.

When considering integration of HIV and CC screening, it is important for policy makers and clinicians to take into account the existing weaknesses and limitations in the health systems. This could be issues to do with availability and skills set of HCP, space (infrastructure), drugs and clinical supplies, linkages to other departments such as laboratories and ART clinic and speed or turnaround time of service delivery. Careful consideration of motivation mechanisms for HCP is equally important. The above areas have to be improved before considering integration of HIV and CC screening.

On the side of the clients, it is important to ensure that effective mechanisms and procedures are in place for booking clinic appointments, minimizing waiting time, pre and post-test counseling women and for addressing HIV stigma both at clinic and community settings. Policy makers and HCP should weigh the cost of fixing the existing weaknesses and limitations in the health system against the benefits of integration of HIV and CC screening as a pre-requisite to integration.

The choice of CC screening algorithm for integration with HIV screening is another critical area for Clinicians to consider. An algorithm that uses HPV testing with self-sampling for triage is most likely going to be faster and will not only reduce on women’s waiting time at the integrated clinic but also reduce on the number of women proceeding to the next stage of CC screening using visual methods or cytology.

Non-mandatory approach to integration with opt-out options for women could be one way of increasing the participation and utilization of the HIV and CC integrated screening services among women who are reluctant to attend clinics where HIV testing is practiced because of fears of HIV stigma. Messages on the opt-out options could be part and parcel of community education outreaches for HIV and CC screening.

And lastly, future research should explore perceptions of HIV and CC screening service integration among community members particularly women, men and community health workers. Such studies will hopefully address questions regarding community acceptability of the HIV and CC care integration in low income country such as Uganda.

## Abbreviations

HIV: Human immune deficiency virus
CC: Cervical cancer
HCP: Healthcare provider
PM: Policy maker
WHO: World health organization
HPV: Human papillomavirus
STIs: Sexually transmitted infections
AIDS: Acquired immune deficiency syndrome
MCH: Maternal child health
RH: Reproductive health
VIA: Visual inspection with 5% acetic acid solution
IRB: Institutional review board
ARV: Antiretroviral drugs
ART: Antiretroviral therapy.
